# Sibutramine as a Cause of Sudden Cardiac Death

**DOI:** 10.1155/2021/8896932

**Published:** 2021-02-06

**Authors:** Miguel Rodriguez-Guerra, Mayank Yadav, Manoj Bhandari, Archna Sinha, Jonathan N. Bella, Eduard Sklyar

**Affiliations:** ^1^BronxCare Health System, New York, USA; ^2^Icahn School of Medicine at Mount Sinai, New York, USA; ^3^St. Charles Hospital, Port Jefferson, New York, USA

## Abstract

*Importance.* Sibutramine was withdrawn from the US market due to association with adverse cardiovascular outcomes especially with patients having preexisting cardiac disease. However, continued presence of sibutramine in herbal medications is a concern to public safety. *Results.* We report a case of a patient with no evidence of previous coronary heart disease, who presented with non-ST elevation acute coronary syndrome (NSTE-ACS). Urgent coronary catheterization showed nonobstructive coronaries. However, patient died unexpectedly within 24 hours of admission. The patient denied toxic habits but was taking over-the-counter weight loss herbal supplements. Complete autopsy was performed which showed sibutramine in the toxicology. Patient died due to complications of acute inadvertent intoxication of sibutramine. *Conclusion and Relevance.* This is the first case report in literature showing association of sibutramine to sudden cardiac death in patients with no prior cardiac history. The continued presence of sibutramine in some over-the-counter weight loss formulations is a very concerning public health issue.

## 1. Introduction

Obesity is one of the modifiable cardiovascular risk factors, and its incidence has increased exponentially over the years. Sibutramine is a combined norepinephrine and serotonin reuptake inhibitor used to reduce appetite and promote weight loss in combination with diet and exercise [1]. At a daily dose of 10–20 mg, it was initially considered to have an excellent safety profile [1, 2]. However, the Sibutramine Cardiovascular Outcomes (SCOUT) trial showed that it has adverse cardiovascular outcomes, especially with patients having preexisting cardiac disease [3]. Based on this report, sibutramine was withdrawn from the US market after the Food and Drug Administration (FDA) recommendations in October 2010. We report a case of a patient who was taking inadvertently sibutramine as a component of over-the-counter weight loss herbal medications who later developed non-ST elevation acute coronary syndrome (NSTE-ACS) and sudden cardiac death.

## 2. Case

A 59-year-old Hispanic female presented to the emergency department (ED) with abdominal pain, nausea, and vomiting that began early in the morning on the day of the presentation. She was drowsy but arousable. She denied any chest pain, shortness of breath, fever, cough, or headache. On further questioning, she denied any history of travel or sick contacts—no history of smoking, drugs, or excessive alcohol use. The patient's medications included benazepril, calcium, alendronic acid, and a “tomato extract herbal supplement” for the weight loss. The previous night she had a vegetable soup before going to sleep and had been in apparently good health until the day of the presentation.

**Figure 1:**
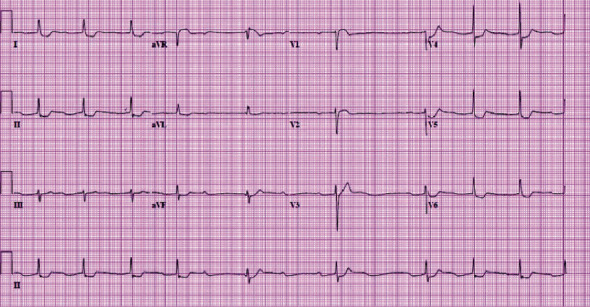
ECG at presentation showed normal sinus rhythm with ST elevation in lead aVR and ST depressions in multiple leads with high-degree atrioventricular (AV) block. ECG: electrocardiogram.

**Figure 2:**
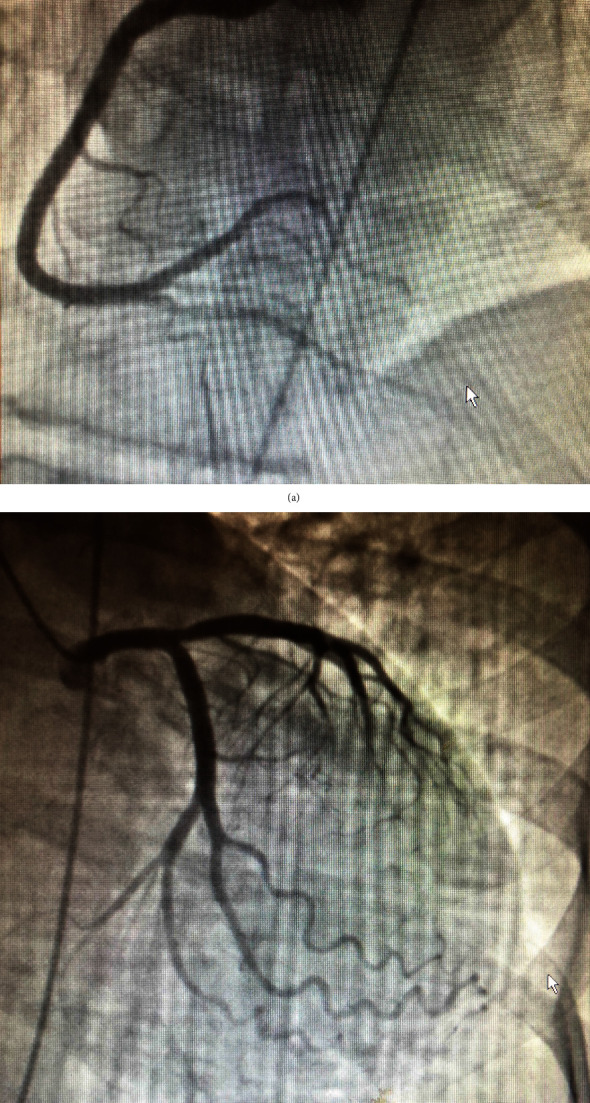
(a) The right coronary angiogram in the left anterior oblique cranial view showing the patent right coronary artery. (b) The left coronary angiogram in the right anterior oblique caudal view showing 50% lesion in the proximal left anterior descending artery, which is not consistent with the presentation as non-ST segment elevation acute coronary syndrome.

On examination, she was afebrile with an irregularly irregular pulse rate of 60/minute, blood pressure of 130/80 mmHg, respiratory rate of 16/minute, and oxygen saturation of 90% at room air, which improved to 95% with 28% oxygen supplementation. Her complete blood count showed leukocytosis of 23.4 cells/dL. The chest X-ray was normal. Electrocardiogram (ECG) showed normal sinus rhythm with ST elevation in lead aVR and ST depressions in multiple leads with high-degree atrioventricular (AV) block (Figure 1). Echocardiogram showed normal left ventricular function and size, with no wall motion abnormalities. The cardiac enzymes, troponin T, and creatinine kinase were elevated, leading to the diagnosis of NSTE-ACS. However, cardiac catheterization showed only 50% stenosis of the proximal left anterior descending artery that could not explain the elevated cardiac enzyme and ECG changes (Figures 2(a) and 2(b)). She was subsequently transferred to the coronary care unit (CCU). In the CCU, the patient was tachypneic with rate 18-22/minute, with an oxygen saturation of 92-94% on 2 liters of oxygen, with a fluctuating mental status. Given elevated leukocyte count of 34.9 cells/dL on repeat complete blood count, broad-spectrum antibiotics (vancomycin, piperacillin-tazobactam, and metronidazole) were started. Computed tomography (CT) scan of the head was normal, and CT abdomen, which also included the lung bases, showed bilateral lower lobe airspace consolidation, possibly pneumonia, and normal finding on the abdominal scan. About 14 hours from her presentation to the hospital, she developed an episode ventricular tachycardia, which reverted spontaneously back to her usual rhythm. Later, she became bradycardic and went into asystole. The patient could not be resuscitated, and she died 15 hours after presentation to the hospital. Because of unexplained death within 24 hours of hospital admission, a complete autopsy was done. At autopsy, there was no evidence of pulmonary embolism or acute myocardial infarction. However, her forensic serum toxicology screen showed sibutramine levels of 0.27 ng/mL, and its metabolized forms of desmethylsibutramine and didesmethylsibutramine were at 0.59 ng/mL and 0.44 ng/mL, respectively. The cause of death was determined as “Complications of Acute Sibutramine Intoxication.”

## 3. Discussion

Sibutramine is a combined norepinephrine and serotonin reuptake inhibitor used as an antiobesity agent to reduce appetite and promote weight loss in combination with diet and exercise [1]. FDA approved it in 1997 in the United States. It initially showed good safety profile [2]. It was shown to improve insulin resistance, glucose metabolism, dyslipidemia, and inflammatory markers, which led to the hypothesis that it may have antiatherogenic properties [1]. However, it also exerts a peripheral sympathomimetic effect, which induces a moderate increase in heart rate and attenuates the reduction in BP attributable to weight loss or even slightly increases BP [1, 4]. It was also shown to prolong QT interval, which could potentially increase the risk of cardiac arrhythmias [1].

Our patient presented with the NSTE-ACS, which on coronary angiography did not show any culprit lesion. The similar presentation was reported by previous case reports [5–7]. All except one case report [8], no coronary lesion was found on coronary angiogram. In all the case reports [5–8], patients were overweight but did not have any comorbidity, including known cardiac disease. However, in the randomized studies, sibutramine was attributed being a high risk of cardiac events only in patients with either known to have cardiac disease or high risk due to comorbidities [3]. The possible explanation for the above presentation can be coronary vasospasm, which is plausible, given the sympathomimetic effects of sibutramine.

We could not find any case report directly attributing sibutramine to sudden cardiac death. Our patient died within 15 hours of presentation and has one episode of ventricular tachycardia, which reverted to normal sinus rhythm. Later, the patient was noted to be bradycardic and hypoxic before demise. The autopsy done did not show anything that could explain the cause of death. However, toxicology studies found significant levels of sibutramine and its metabolites desmethylsibutramine and didesmethylsibutramine. As per the autopsy report, the cause of death was determined as “Complications of Acute Sibutramine Intoxication.”

Since 2002, several cardiovascular adverse events (hypertension, tachycardia, arrhythmias, and myocardial infarction) were reported in sibutramine-treated patients. This led to SCOUT trial evaluating the long-term effects of sibutramine treatment combined with diet and exercise on the rates of cardiovascular events and cardiovascular death among subjects who were at high cardiovascular risk [3]. It showed that in patients with preexisting cardiovascular conditions receiving long-term sibutramine treatment, there was an increased risk of nonfatal myocardial infarction and nonfatal stroke but not of cardiovascular death or death from any cause [3]. Because the benefit of sibutramine as a weight loss drug seems not to outweigh the cardiovascular risks, it was withdrawn from the US and European markets. However, the continued presence of sibutramine in some over-the-counter weight loss formulations is a very concerning public health issue. The FDA has now implemented stricter guidelines for the control of dietary supplements, which is a welcome step to ensure public safety [10]. Aggressive internet-based marketing plays a vital role in capturing customers for over-the-counter supplements; unfortunately, despite its withdrawal from the market since 2010, sibutramine continues to be present in many antiobesity food supplements sold on the Internet [11]. The current FDA website search for sibutramine mentions 250 results of public notice of the supplements which contain sibutramine. Still, one recent study showed that 66.7% of recalled supplements still available for purchase at least six months after FDA recalls remained adulterated with banned ingredients [12]. Further investigations are as needed to determine the effectiveness of policies on public safety, given the continued presence of some banned substances in some dietary supplements.

## 4. Conclusion

This case, though unfortunate eventual outcome, has few unique aspects that are worth reporting and sharing, so that knowledge thus gained can help to save other patients. First, to the best of our knowledge, this is the first case report showing an association of sibutramine with sudden cardiac death. Second, it is the sixth case report in PubMed in a patient with no medical comorbidities taking sibutramine that presented with NSTE-ACS [5–9]. Third, it is the concerning fact that the drug is still available to the patient as a component of some herbal weight reduction supplements despite being withdrawn from the market [5, 6].

## Data Availability

Data sharing is not applicable to this article as no data sets were generated. The actual information are available in MR system of our hospital and from the corresponding author with reasonable request.
